# Parental Use of Corporal Punishment in Europe: Intersection between Public Health and Policy

**DOI:** 10.1371/journal.pone.0118059

**Published:** 2015-02-12

**Authors:** Nathalie duRivage, Katherine Keyes, Emmanuelle Leray, Ondine Pez, Adina Bitfoi, Ceren Koç, Dietmar Goelitz, Rowella Kuijpers, Sigita Lesinskiene, Zlatka Mihova, Roy Otten, Christophe Fermanian, Viviane Kovess-Masfety

**Affiliations:** 1 Mailman School of Public Health, Columbia University, Department of Epidemiology, New York, New York, United States of America; 2 École des Hautes Études en Santé Publique, EA4057 Paris Descartes University, Paris, France; 3 The Romanian League for Mental Health, Bucharest, Romania; 4 Yeniden Health and Education Society, Istanbul, Turkey; 5 Institute of Psychology, University of Koblenz-Landau Campus, Koblenz, Germany; 6 Clinic of Psychiatry, Faculty of Medicine, University of Vilnius, Vilnius, Lithuania; 7 New Bulgarian University, Sofia, Bulgaria; 8 Behavioural Science Institute, Radboud University Nijmegen, The Netherlands; Georgia State University, UNITED STATES

## Abstract

Studies have linked the use of corporal punishment of children to the development of mental health disorders. Despite the recommendation of international governing bodies for a complete ban of the practice, there is little European data available on the effects of corporal punishment on mental health and the influence of laws banning corporal punishment. Using data from the School Children Mental Health Europe survey, the objective of this cross-sectional study was to examine the prevalence and legal status of corporal punishment across six European countries and to evaluate the association between parental use of corporal punishment and children’s mental health. The study found that odds of having parents who reported using occasional to frequent corporal punishment were 1.7 times higher in countries where its use is legal, controlling for socio-demographic factors. Children with parents who reported using corporal punishment had higher rates of both externalized and internalized mental health disorders.

## Introduction

Countries, and individuals within countries, vary greatly on normative approaches to child rearing; however, the debate over the use of non-abusive physical punishment (corporal punishment) across many countries is growing. The American Academy of Pediatrics (AAP) defines corporal punishment as the application of physical pain, such as spanking, slapping, or grabbing to abate an undesirable child behavior [1]. Corporal punishment remains legal and relatively well-accepted form of child discipline in the United States (US), with prevalence studies reporting 64–95% of parents use spanking between the ages of 2–3 [2]. However a growing number of countries are passing laws prohibiting its use within the home [3]. International governing bodies including the United Nations (UN) and the European Union (EU) have become strong advocates for a child’s right to a non-violent upbringing, and recommended member states enact legislation banning all forms of corporal punishment [4]. As of 2012, twenty-three of forty-seven Council of Europe (CoE) countries have passed laws prohibiting the use of corporal punishment within the home [5]. Despite the growing momentum of policy initiatives to end corporal punishment of children, the long-term health consequences of corporal punishment remains a topic of debate [3].

Corporal punishment has been shown to be effective in stopping or preventing the undesired behavior in the short-term [3]. However, the possibility of negative long-term effects on children’s behavioral and mental health has long been a concern within the medical and psychological communities [3]. A number of studies have demonstrated an association between corporal punishment and a variety mental health and behavioral problems [6,7,8]. A 2002 meta-analysis of the relationship between corporal punishment and adverse mental health outcomes found that corporal punishment was associated with eleven important childhood behaviors and experiences including increased aggression and anti-social behavior [3].

A great breadth of research has been conducted within the US demonstrating the adverse effects of corporal punishment. Internationally, the social acceptability and prevalence of corporal punishment differs greatly across country [9]. Parents attitudes towards harsh punishment have been shown to be predictive of future use of harsh punishment [10], and while cultural norms shape parental opinions on the use of corporal punishment [11], these norms are not necessarily immutable. Studies conducted in Sweden and Finland, the first two countries to ban corporal punishment in 1979 and 1983 respectively, have shown significant decrease in support for corporal punishment, as well as, a decline in prevalence [12,13]. The School Children Mental Health Europe (SCMHE) project provides a unique opportunity to study the use of corporal punishment and it’s relation to children’s mental health problems on an international scale. Conducted to assess the overall state of mental health of European children, the survey includes data from six different countries including: Bulgaria, Germany, Lithuania, the Netherlands, Romania, and Turkey. The present study examines the association between corporal punishment and children’s mental health across these diverse contexts and cultures. Additionally, we explored potential associations between legislation banning corporal punishment on the frequency of parental corporal punishment, and the relation between corporal punishment and child mental health.

## Methods

### Study Population/Data Source

The SCMHE survey, a cross sectional study conducted in 2010, evaluated the mental health of school children aged 6 to 11 across Europe. Data for the present study was taken from six countries where parental use of corporal punishment was measured: Bulgaria (n = 1004), Germany (n = 471), Lithuania (n = 1112), the Netherlands (n = 671), Romania (n = 1121), and Turkey (n = 578) for a total of 4,957 children. In each participating country, approximately 45 to 50 grade schools were randomly selected and approached. Participation rates at the school level varied between 6.5% (Netherlands) and 95.6% (Romania). Classes were randomly selected in each school, and approximately six children were randomly selected in each class. Approximately 48 children were selected from each school, except in the Netherlands where more children were selected from each school because a lesser number of schools participated. Children of parents who failed to answer the question related to frequency of corporal punishment were excluded. The percentage of parents answering the corporal punishment question varied by country: Bulgaria 72%, Germany 59%, Lithuania 87%, The Netherlands 45%, Romania 80%, and Turkey 63%. Teachers of students selected to participate were surveyed using paper questionnaires with questions concerning the child’s behavior and emotional state. Parents received an informational letter and consent form to be returned to the school. If the parent did not return the consent form, stating their refusal to participate, the child was included. The child’s parent, either mother or father, were queried using a paper based demographic and social questionnaire concerning household composition, parental education, occupational data, unemployment status, marital status, and questions concerning the parent/child relationship. In the Netherlands the same questions were made available electronically using a secured website, although paper copies were available upon request. Additional information about sampling methods was included in the final SCMHE report [14].

### Measures

**Fig 1:**
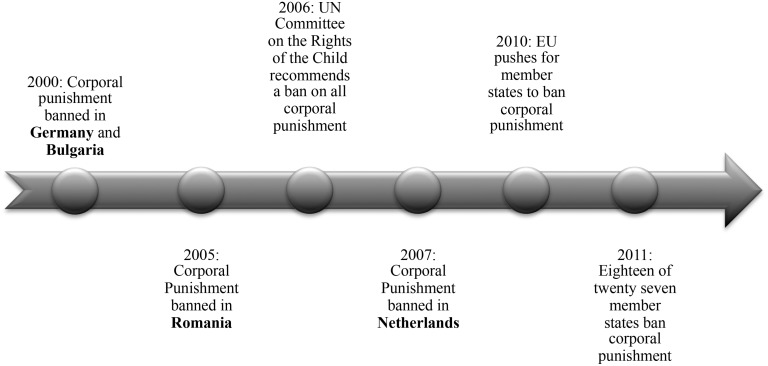
Timeline of corporal punishment policies. Including international recommendations and country specific legislation.

**Legality of Corporal Punishment**. The legal status of corporal punishment was assessed using country reports issued by the Global Initiative to End All Corporal Punishment of Children [15]. At the time of the study all but two of the countries, Turkey and Lithuania, had a legal ban prohibiting the use of corporal punishment in the home. Fig. 1 provides a timeline of country-specific corporal punishment legislation and recommendations for the banning of corporal punishment by international governing bodies.

**Parental Use of Corporal Punishment**. The parental questionnaire included questions regarding the relationship of the parent and the child. It included a question on the frequency of corporal punishment within the home, “When my child misbehaves I spank, slap, grab, or hit my child.” Responses were on a scale from 0 to 6 with 0 being never/rarely and 6 being always/most of the time (S1 Table for a distribution of responses by country). Because of the distribution of the data the variable was collapsed into a binary variable (0–2 considered infrequent corporal punishment, 3–6 considered frequent). We also analyzed frequency of corporal punishment as a continuous variable in a sensitivity analysis.

**Child’s Mental Health**. Child mental health data was collected from three informants: the child, the parent, and the teacher. Child report was examined using the extensively validated instrument the Dominic Interactive (DI) [16–19]. The DI is self-administered computer-based questionnaire developed for children ages 6–11. Children follow a cartoon character matched to the child’s race and gender through 91 questions designed to determine the likely presence or absence of three external psychopathologies, oppositional defiant, conduct disorder, and ADHD, and four internal psychopathologies, separation anxiety, phobia, general anxiety, and depression. Child psychopathology was also evaluated by the previously validated parent and teacher Strengths and Difficulties Questionnaire (SDQ). The parent and teacher versions of the SDQ include a brief questionnaire divided into 5 subscales: emotional problems, hyperactivity and inattention, conduct problems, peer relationship difficulties and prosocial behaviours [20, 21].

**Covariates**. The covariates mother’s age, father’s age, reporting parent’s gender, highest level of parental education, parental occupation, unemployment, patent marital status, and single parent household were included in the analysis based on a priori research suggesting a link between these socio-demographic factors and parental use of corporal punishment [22].

### Ethics Statement

Each participant received a written informed consent form for refusal to be signed and returned to the school, describing the survey, anonymity protections, the formal responsible authorities and their contacts and the aims of the study.

All participating countries had the support of their governments, including their ministers of education and health and received ethical approval from the corresponding authority (Bulgaria: Republic of Bulgaria, Deputy Minister of Education, Youth and Science; Germany: Ministry of Education, Science and Culture, Mecklenburg-Vorpommern, State school authority, Luneburg, Ministry of Education and Culture of Schleswig-Holstein country; Italy: Ethical Committee of the Association of European University Mediterranean ONLUS; Lithuania: Republic of Lithuania—Ministry of Education and Science; Netherlands: Commission of Faculty Ethical Behavior Research (ECG); Romania: Bucharest School Inspectorate General Municipal; Turkey: Istanbul—directorate of National Education). Data were collected without any names and therefore analyzed anonymously.

### Statistical Analysis

First, the prevalence of parents reporting using corporal punishment was assessed both as a seven level categorical variable (response options 0 through 6 on frequency of corporal punishment item, with 0 as the reference group) and as a binary variable (0–2 versus 3–6). Second, the socio-demographic factors associated with parental report of corporal punishment were explored using descriptive statistics and chi-square tests. Third, we analyzed the relationship between parental report of corporal punishment and children’s mental health problems using chi-squared tests. Fourth, we estimated the association between parental report of corporal punishment and the child’s mental health problems stratified by whether the country of residence of the respondent has a policy regarding the legal status of corporal punishment. Odds ratios and 95% confidence intervals were estimated. Finally, the interaction between use of corporal punishment and the legality of corporal punishment was assessed with cross-product terms in an interaction model; F-tests were calculated to estimate the statistical significance of inclusion of the interaction term. All analyses were done using SAS 9.1.

## Results

### Socio-demographic factors and parental use of corporal punishment

Table 1 shows the differences in socio-demographic characteristics between parents who reported using frequent corporal punishment and those who did not report using frequent corporal punishment. Those who reported using corporal punishment were on average younger (p-value <0.0001), less educated (p-value <0.0001), and were less likely to be employed in professional and management jobs (p-value <0.0001). They also tended to have higher rates of unemployment (p-value <0.0001). There was no association between marital status of mothers and father (p-values 0.1462, 0.8827) or being a single parent (p-value 0.1148) and reporting using frequent corporal punishment.

**Table 1:** Socio-demographic factors comparing those children whose parents reported using corporal punishment to those children whose parents did not report using corporal punishment.

Social Determinant	Frequent corporal punishment %(N)	Infrequent corporal punishment %(N)	Chi-Sq (p-value)
Mother Age			**29.7 (<.0001)**
<35	57.9 (245)	44.1 (1910)	
35–40	24.1 (102)	31.0 (1343)	
>40	18.0 (76)	24.9 (1078)	
Father Age			**20.1 (<.0001)**
<35	40.9 (148)	30.3 (1195)	
35–40	31.5 (114)	32.9 (1299)	
>40	27.6 (100)	36.9 (1456)	
Highest level of education both parents			**25.3 (<.0001)**
Some primary or secondary	17.4 (69)	9.8 (416)	
Secondary Completed	38.8 (154)	37.6 (1593)	
College or technical school completed	43.8 (174)	52.6 (2228)	
Unemployment			**18.8 (<.0001)**
Both parents employed	77.3 (262)	85.4 (2769)	
One parent unemployed	18.6 (63)	12.8 (415)	
Both parents unemployed	4.1 (14)	1.8 (57)	

### Association between parental use of corporal punishment and countries legal status of corporal punishment

Table 2 shows the number of parents, by country, reporting using frequent vs. infrequent corporal punishment when their child misbehaves. The two countries where corporal punishment is still legal, Lithuania and Turkey, had the highest percentage of parents reporting using physical punishment 12.7% and 11.6% respectively. Table 3 shows the relationship between legal status of corporal punishment and parental reporting of frequent use of corporal punishment. After controlling for mother and father age, parental education level, and parental unemployment the odds of reporting frequent corporal punishment was 1.7 times higher (95% CI 1.37–2.21) in countries where its use remains legal.

**Table 2:** Parent’s response by country to the question “when my child misbehaves I hit, spank, or slap my child” as a binary variable.

Country (total n)	Parental Response % (n)	
	Infrequent corporal punishment*	Frequent corporal punishment*
Bulgaria (1004)	90.9(913)	9.1(91)
Germany (471)	96.6 (455)	3.4(16)
Lithuania (1112)	87.3(971)	12.7(141)
Netherlands (671)	97.0(651)	3.0(20)
Romania (1121)	90.7(1017)	9.3(104)
Turkey (578)	88.4(511)	11.6(67)
**Total (4957)**	**91.1(4518)**	**8.9(439)**

*Infrequent corporal punishment defined as 0–2 on frequency of corporal punishment item; frequent defined as 3–6.

**Table 3:** Association between legal status of corporal punishment and parental report of frequent use of corporal punishment.

	Odds Ratios	95% Confidence Intervals	P-value
Unadjusted	1.9	(1.5–2.3)	<0.0001
Adjusted*	1.7	(1.4–2.2)	<0.0001

* Adjusted model controlled for mother and father age, parental education level, and parental unemployment

### Association between parental use of corporal punishment and children psychopathology

Table 4 compares the prevalence of children’s mental health problems, as determined by the Dominic Interactive (DI) and parent and teacher Strengths and Difficulties Questionnaire (SDQ) between parents reporting little to no use of corporal punishment and those reporting frequent use of corporal punishment. In the child report, the rates of any externalized problems were significantly higher in children whose parents reported frequent use of corporal punishment 13.4% compared to 6.3% (p-value <0.0001) Rates of internalized problems were also higher in those children whose parents reporting frequent corporal punishment 23.3% compared to 18.7% (p-value 0.0268). All three externalized problems were significantly higher in children whose parents reported frequent use of corporal punishment, most notably oppositional defiant disorder, which was identified in 7.5% of children whose parents reported frequent use of physical punishment compared to 3.3% of children whose parents reported using corporal punishment infrequently (p-value <0.0001). While separation anxiety and phobia were not associated with parental use of corporal punishment, there was a significant association between corporal punishment and depression and anxiety (p 0.003 and 0.0018). Both parents and teachers reported significantly higher rates of any externalized disorder: 40.4% compared to 17.2% (p-value <.0001) and 32.0% compared to 19.3%(p-value <.0001) respectively. Emotional problems were significantly different between the groups on parent report, but not on teacher report (p-values 0.0047 and p 0.3902 respectively).

**Table 4:** Prevalence of externalized and internalized mental health disorders comparing prevalence between those whose parents report using physical punishment and those who report no or limited use of physical punishment using three informants child, parent, and teacher.

Mental Disorder Prevalence	Frequent corporal punishment %(N)	Infrequent corporal punishment %(N)	P-value
**Child DI**			
*Any External*	*13.4 (57)*	*6.3 (280)*	*<.0001*
Oppositional Defiant	7.5 (32)	3.3 (148)	<.0001
Conduct Disorder	5.4 (23)	3.2 (143)	.0171
ADHD	5.4 (23)	3.0 (135)	.0082
*Any Internal*	*23.3 (99)*	*18.7 (839)*	.*0268*
Separation Anxiety	13.4 (57)	12.4 (553)	.5600
Phobia	8.9 (38)	6.4 (284)	.0427
General Anxiety	8.0 (34)	4.6 (204)	.0018
Depression	7.5 (32)	3.8 (171)	.0003
**Parent SDQ**			
*Any External*	*40.4 (177)*	*17.2 (849)*	*<.0001*
Conduct Problems	30.1 (132)	11.8 (531)	<.0001
Hyper Activity	24.0 (105)	11.4 (511)	<.0001
Peer Relationship Issues	33.1 (145)	25.1 (1,131)	.0003
*Any Internal*	*20.8 (91)*	*15.6 (701)*	.*0047*
Emotional Problems	20.8 (91)	15.6 (701)	.0047
**Teacher SDQ**			
*Any External*	*32.0 (135)*	*19.3(849)*	*<.0001*
Conduct Problems	26.8 (113)	13.6 (570)	<.0001
Hyper Activity	22.8 (96)	12.3 (517)	<.0001
Peer Relationship Issues	11.6 (49)	9.8 (411)	.2363
*Any Internal*	*6.9 (29)*	*8.1 (338)*	.*3902*
Emotional Problems	6.9 (29)	8.1 (338)	.3902

Tables 5 and 6 provide logistic regression models demonstrating the association between parental report of frequent use of corporal punishment and children’s mental health problems as determined by the child DI with and stratified on legal status of corporal punishment. Odds ratios for any and all externalized problems were significant in countries where corporal punishment remains legal. In those countries where corporal punishment is illegal any external and oppositional defiant disorder remained significant but had lower odds ratios. The interaction between use of corporal punishment and legal status of the country was statistically significant for conduct disorder as an outcome (p-value 0.03). Among those in countries where corporal punishment is illegal, children of parents who use corporal punishment had 2.6 times the odds of reporting conduct problems (95% C.I. 1.5–4.5); among those in countries where corporal punishment is legal, children of parents who use corporal punishment had 0.9 times the odds of reporting conduct problems (95% C.I. 0.4–2.0). For internalized problems, the odds ratios for any internalized problem, general anxiety, and depression were significant in countries where corporal punishment is illegal and no internalized problems were significant in countries where corporal punishment is legal. The interaction between use of corporal punishment and legal status of the country trended towards statistical significance for depression as an outcome (p-value 0.06). Among those in countries where corporal punishment is illegal, offspring of those who use corporal punishment had 2.8 times the odds of depression (95% C.I. 1.6–4.7); among those in countries where corporal punishment is legal, offspring of those who use corporal punishment had 1.3 times the odds of conduct disorder (95% C.I. 0.7–2.3).

**Table 5:** Association between parental report of frequent use of corporal punishment and children’s external mental health disorders as determined by the child DI stratified by legal status of corporal punishment.

	**Corporal Punishment Illegal**			**Corporal Punishment Legal**		
	Odds Ratios	95% Confidence Intervals	P-value	Odds Ratios	95% Confidence Intervals	P-value
Any External	2.7	(1.8–4.0)	<0.0001	2.0	(1.2–3.2)	0.0069
Oppositional Defiant	2.8	(1.8–4.5)	<0.0001	2.2	(1.1–4.6)	0.0302
Conduct Disorder	2.6	(1.5–4.5)	0.0005	0.85	(0.36–2.0)	0.7164
ADHD	1.9	(1.1–3.5)	0.0335	1.8	(0.90–3.7)	0.0961

**Table 6:** Association between parental report of frequent use of corporal punishment and children’s internal mental health disorders as determined by the child DI stratified by legal status of corporal punishment.

	**Corporal Punishment Illegal**			**Corporal Punishment Legal**		
	Odds Ratios	95% Confidence Intervals	P-value	Odds Ratios	95% Confidence Intervals	P-value
Any Internal	1.5	(1.1–2.0)	0.0194	1.1	(0.78–1.6)	0.5804
Separation Anxiety	1.2	(0.84–1.8)	0.2929	1.0	(0.62–1.6)	0.9599
Phobia	1.4	(0.85–2.3)	0.1875	1.4	(0.84–2.3)	0.2034
General Anxiety	2.2	(1.3–3.5)	0.0030	1.4	(0.79–2.5)	0.2469
Depression	2.8	(1.6–4.7)	0.0001	1.3	(0.72–2.3)	0.3784

Finally, in a sensitivity analysis we examined frequency of corporal punishment as a continuous independent variable in the logistic regression models. Results are provided in S2 Table (parent report of child mental health) and S3 Table (child report of his/her own mental health). Results were consistent with the main analysis; there was an association between frequency of corporal punishment and increased odds of child mental health, especially among those in countries where corporal punishment is illegal. In fact, for child reported mental health outcomes (S3 Table), there were no associations between frequency of corporal punishment and odds of mental health problems among those in countries where corporal punishment is legal, whereas there were consistent significant associations among those in countries where corporal punishment is illegal.

## Discussion

The present study of more than 4,000 European school children across six countries documents that corporal punishment is associated with adverse mental health outcomes in children, and that the association between corporal punishment and adverse mental health is stronger among children in countries that have explicit policies banning the practice. These data are consistent with prior US studies documenting a correlation between corporal punishment and mental health problems, including prior studies documenting increased aggression and other externalized problems [23,24], as well as an increase in childhood depression and anxiety, and, later in life, suicidal tendency [7,25]. The present study extends this previous literature by confirming the association in a diverse international context. The congruency of this new European data with prior US studies, suggests that the negative effects of corporal punishment are consistent across a wide range of cultural contexts.

The SCMHE data also supports prior study findings such as Berlin et al., that socio-demographic factors such as young age, less education and higher rates of unemployment are all associated with an increase in parental use of corporal punishment [26]. However, even when considering socio-demographic factors associated with corporal punishment, the SCHME data found an association between parental use of corporal punishment and the legal status of corporal punishment within countries.

Our data indicate that the prevalence of corporal punishment is highest in Turkey and Lithuania, the two countries where its use remains legal. Additionally, multivariable analysis found a positive association between legality of corporal punishment and frequency of parental use of corporal punishment, even after adjustment for socio-demographic factors. Currently, only eighteen of twenty-seven EU member states have any legislation banning the use of corporal punishment in the home [5]. The cross-sectional design of the present study precludes longitudinal assessment of whether changes in laws predict changes in behavior, thus it is possible that cultural views changed and corporal punishment became more socially unacceptable prior to legislation. However, this study provides evidence that corporal punishment is negatively associated with children’s mental health, and that the absence of legislation banning corporal punishment is associated with greater parental use of corporal punishment. Countries should revaluate and move forward with the EU and UN recommended bans on corporal punishment given the results of this study.

Study limitations are noted. While children were selected using several levels of randomization (school, class, student), some were excluded, because not all parents answered the corporal punishment question, which may limit generalizability. Participation rates of parents answering the question regarding corporal punishment were lowest in the Netherlands and Germany 45% and 59% respectively. These countries also reported the lowest rates of corporal punishment (3% and 3.4%). It is possible that parents underreported in these countries, however response rates were also low in Turkey (63%), a country that had one of the highest percentage of parents reporting frequent use of corporal punishment (11.6%), suggesting response rates may be due to other unknown factors. Further, measurement of child psychopathology based on child, parent, and teacher report and not clinical diagnoses by a trained professional. However, the Dominic Interactive and SDQ are well-validated [16–19], and the relationship between children’s mental health and frequent and infrequent corporal punishment is consistent across informants (child, parent, and teacher). It is also possible that the differences in corporal punishment between counties where its practice is legal versus where it is legal could be the result of an unknown cultural confounder. The cross-sectional nature of the study provides only a snap shot of parental views on corporal punishment and it is not possible to account for any shifts in cultural acceptance before and after legalization.

## Conclusions

Despite these limitations, the SCMHE data provided an excellent opportunity to begin to examine the relationship between legality and prevalence of corporal punishment, as well as the association between corporal punishment and children’s mental health disorders within a diverse European context. The study has provided additional evidence of the negative impact of corporal punishment on children’s mental health, and provided preliminary evidence that countries with no legal ramifications are more likely to have parents whom engage in the practice of corporal punishment. Additional studies are warranted that specifically look at legality, parental perception and use of corporal punishment and their relationship to children’s mental health.

## Supporting Information

S1 Table Parent’s response by country to the question “when my child misbehaves I hit, spank, or slap my child” as a seven level categorical variable. (DOCX)

S2 Table Association between parental report of frequent use of corporal punishment and children’s external mental health disorders as determined by the child DI stratified by legal status of corporal punishment where corporal punishment is a seven level categorical variable. (DOCX)

S3 Table Association between parental report of frequent use of corporal punishment and children’s internal mental health disorders as determined by the child DI stratified by legal status of corporal punishment where corporal punishment is a seven level categorical variable. (DOCX)
