# Supplementary material for: Parental Use of Corporal Punishment in Europe: Intersection between Public Health and Policy
Source: PLoS One. 2015 Feb 12;10(2):e0118059. doi: 10.1371/journal.pone.0118059 (PMC4326463; doi:10.1371/journal.pone.0118059)
Supplement: S1 Table — (DOCX) [file pone.0118059.s001.docx]

|  | | | | | | | | | | |
| --- | --- | --- | --- | --- | --- | --- | --- | --- | --- | --- |
| Country (n) | Parental Response Percentage (n) | | | | | | | | | |
|  | **Never or Rarely (0)** | **1** | **2** | **3** | **4** | **5** | | **Most of the Time (6)** |  |  |
| Bulgaria (1004) | 62.9 (632) | 18.8 (188) | 9.3 (93) | 5.4(54) | 0.8(8) | 1.0(10) | 1.9 (19) | | |  |
| Germany (471) | 77.1 (363) | 16.1(76) | 3.4 (16) | 2.3 (11) | 0.4 (2) | 0.4 (2) | 0.2 (1) | | |  |
| Lithuania (1112) | 48.8 (543) | 22.6 (251) | 15.9 (177) | 7.1 (79) | 2.3 (26) | 1.1 (12) | 2.2 (24) | | |  |
| Netherlands (671) | 71.1 (477) | 22.7 (152) | 3.3 (22) | 1.9 (13) | 1.0 (7) | - | - | | |  |
| Romania (1121) | - | 75.5 (846) | 15.3 (171) | 4.7 (53) | 1.0 (11) | 0.9 (10) | 2.7 (30) | | |  |
| Turkey (578) | 57.3 (331) | 19.0 (110) | 12.2 (70) | 5.1 (30) | 1.6 (9) | 1.0 (6) | 3.8 (22) | | |  |
| **Total (4957)** | **47.3 (2346)** | **32.7 (1,623)** | **11.0 (549)** | **5.0 (240)** | **1.3 (63)** | **3.8 (22)** | **1.9 (96)** | | |  |
