# Supplementary material for: Parental Use of Corporal Punishment in Europe: Intersection between Public Health and Policy
Source: PLoS One. 2015 Feb 12;10(2):e0118059. doi: 10.1371/journal.pone.0118059 (PMC4326463; doi:10.1371/journal.pone.0118059)
Supplement: S2 Table — (DOCX) [file pone.0118059.s002.docx]

|  | **Corporal Punishment Illegal** | | | **Corporal Punishment Legal** | | |
| --- | --- | --- | --- | --- | --- | --- |
|  | Odds Ratios | 95% Confidence Intervals | P-value | Odds Ratios | 95% Confidence Intervals | P-value |
| Any External | 1.2 | (1.1-1.4) | <0.0001 | 1.1 | (1.0-1.3) | 0.0709 |
| Oppositional Defiant | 1.2 | (1.1-1.4) | 0.0012 | 1.2 | (1.0-1.4) | 0.0477 |
| Conduct Disorder* | 1.3 | (1.2-1.3) | 0.0007 | 1.0 | (0.9-1.1) | 0.7096 |
| ADHD | 1.1 | (1.0-1.3) | 0.1124 | 1.1 | (0.9-1.3) | 0.5988 |

*Significant interaction term included in the model (p=0.0282)
