# Supplementary material for: Parental Use of Corporal Punishment in Europe: Intersection between Public Health and Policy
Source: PLoS One. 2015 Feb 12;10(2):e0118059. doi: 10.1371/journal.pone.0118059 (PMC4326463; doi:10.1371/journal.pone.0118059)
Supplement: S3 Table — (DOCX) [file pone.0118059.s003.docx]

|  | **Corporal Punishment Illegal** | | | **Corporal Punishment Legal** | | |
| --- | --- | --- | --- | --- | --- | --- |
|  | Odds Ratios | 95% Confidence Intervals | P-value | Odds Ratios | 95% Confidence Intervals | P-value |
| Any Internal | 1.1 | (1.1-1.2) | 0.0006 | 1.0 | (1.0-1.1) | 0.5486 |
| Separation Anxiety | 1.1 | (1.0-1.2) | 0.0040 | 1.0 | (0.9-1.1) | 0.9321 |
| Phobia | 1.1 | (1.0-1.3) | 0.0293 | 1.1 | (1.0-1.2) | 0.2516 |
| General Anxiety | 1.2 | (1.0-1.3) | 0.0334 | 1.0 | (0.9-1.2) | 0.5812 |
| Depression | 1.2 | (1.0-1.4) | 0.0083 | 1.1 | (0.9-1.2) | 0.3104 |
